# Histone demethylase JMJD2D promotes macrophage IL-12b expression and contributes to host defense against *Listeria monocytogenes* infection

**DOI:** 10.3389/fcimb.2026.1901735

**Published:** 2026-09-30

**Authors:** Yilin Hong, Hanshi Guo, Bei Li, Jing Jin, Yang Zhao, Junjie Zhang, Jingna Yuan, Xiaochun Xia, Chundong Yu

**Affiliations:** 1 Xiamen Key Laboratory of Traditional Chinese Medicine Bio-engineering, School of Pharmacy, Xiamen Medical College, Xiamen, Fujian, China; 2 State Key Laboratory of Cellular Stress Biology, Innovation Center for Cell Biology, School of Life Sciences, Xiamen University, Xiamen, Fujian, China; 3 Ordos Center for Disease Control and Prevention, Ordos, China; 4 Department of Public Health and Medical Technology, Xiamen Medical College, Xiamen, Fujian, China

**Keywords:** histone demethylation, IFNγ, IL-12b, innate and adaptive immunity, JMJD2D, *Listeria monocytogenes*

## Abstract

**Introduction:**

*Listeria monocytogenes* (*L. monocytogenes*) is an intracellular bacterial pathogen that requires coordinated innate and adaptive immune responses for effective clearance. However, the epigenetic mechanisms governing host defense against *L. monocytogenes* remain poorly understood.

**Methods:**

JMJD2D-deficient mice and wild-type littermates were infected with *L. monocytogenes* to assess host susceptibility, bacterial burdens, tissue damage, and survival. Bone marrow-derived macrophages (BMDMs) and peritoneal macrophages (PMs) were isolated for *in vitro* infection assays. Molecular mechanisms were investigated in macrophages using siRNA-mediated knockdown, luciferase reporter assays, co-immunoprecipitation (Co-IP) and chromatin immunoprecipitation (ChIP).

**Results:**

JMJD2D deficiency markedly increased host susceptibility to *L. monocytogenes* infection, resulting in elevated bacterial burdens, aggravated tissue damage, and reduced survival. Mechanistically, JMJD2D promoted NOD2-associated innate sensing and enhanced IL-12b transcription, accompanied by reduced H3K9me3 enrichment at the IL-12b promoter and cooperation with c-Rel. JMJD2D deficiency was associated with reduced IL-12p70 secretion and impaired macrophage bactericidal activity. Furthermore, JMJD2D contributed to coordinated macrophage and CD8^+^ T cell responses, as JMJD2D promoted IL-12b expression and IL-12p70 production in macrophages, while IFNγ enhanced macrophage IL-12b expression through the IFNGR2-JAK2-STAT1 pathway. Notably, the catalytic activity of JMJD2D contributed substantially to the full induction of *L. monocytogenes*-induced IL-12b expression and inflammatory responses in macrophages.

**Discussion:**

These findings identify JMJD2D as an epigenetic regulator of macrophage IL-12b expression and IL-12p70 production, and support its involvement in coordinated macrophage and CD8^+^ T cell responses during *L. monocytogenes* infection. Our findings further suggest that the JMJD2D-IL-12b/IL-12p70-IFNγ regulatory axis contributes to host antimicrobial immunity and provide a basis for further investigation of JMJD2D as a potential modulator of antibacterial immune responses.

## 1 Introduction

*Listeria monocytogenes* (*L. monocytogenes*) is an opportunistic intracellular bacterium that can cause listeriosis in humans, with a mortality rate up to 20%-30% (Meireles et al., 2024). Following the ingestion of food contaminated with *L. monocytogenes*, the bacteria breaches the intestinal epithelial barrier into the lamina propria, disseminates to the liver and spleen via the lymphatic and blood systems, and exhibits a marked tropism for crossing the blood-brain and placental barriers in immunocompromised individuals and pregnant women, respectively (Cossart, 2011; Ireton et al., 2021). *L. monocytogenes* infection poses a particularly grave threat to the elderly, pregnant women, unborn babies, and persons with impaired immune systems (*e.g.*, those with AIDS, cancer, or transplant recipients) (Osek and Wieczorek, 2022), often leading to miscarriage or severe neonatal complications (Li et al., 2020a). Thus, understanding the host immune mechanisms against *L. monocytogenes* is important for public health.

The innate immune system serves as the first and crucial line of defense against *L. monocytogenes*, with macrophages serving as the primary cells responsible for its clearance (Emoto et al., 2010; Garcia-Diaz et al., 2017). Upon recognizing *L. monocytogenes*, macrophages activate inflammatory responses through various pathogen pattern recognition receptors including NOD2, that help control the infection (Witte et al., 2012; Lee et al., 2023). However, *L. monocytogenes* secretes virulence factors that facilitate phagosomal escape, enabling the pathogen to avoid degradation and replicate in the host cytoplasm (Parihar et al., 2013; Li et al., 2024). To overcome this evasion, the innate immune response triggers a targeted adaptive immunity. The key initiator is IL-12p70, a heterodimeric cytokine composed of IL-12a (p35) and IL-12b (p40) subunits, which is produced upon detection of intracellular bacteria and drives the differentiation of pathogen-specific T-helper 1 (Th1) cells (Hashiguchi et al., 2014). These Th1 cells then secrete high levels of interferon-gamma (IFNγ), a potent macrophage-activating cytokine (Eshleman et al., 2017; Nakane et al., 2025). Notably, T cell-derived IFNγ production is largely dependent on IL-12 (Glassman et al., 2021), highlighting the pivotal role of IL-12 in bridging innate and adaptive immunity. Once activated by IFNγ, macrophages potentiate their antimicrobial mechanisms, including the production of reactive oxygen species (ROS), nitric oxide (NO), and other mediators, thereby inhibiting bacterial phagosomal escape and cytoplasmic replication (Xia et al., 2021; Zhai et al., 2025). This establishes a positive feedback loop wherein innate immune signals instruct the adaptive response, which in turn amplifies the innate killing capacity of macrophages to ultimately control the infection.

Jumonji C domain-containing histone demethylase 2D (JMJD2D, also known as KDM4D) is an epigenetic regulator that catalyzes the demethylation of repressive histone marks H3K9me2/3 to activate gene transcription (Chen et al., 2022). Initially characterized as a driver of cancer progression through diverse mechanisms, including Wnt/β-catenin activation (Peng et al., 2019), p53 antagonism (Li et al., 2020b), and HIF1-mediated glycolysis (Peng et al., 2020), JMJD2D also exhibits critical, context-dependent functions during infection. It is exploited by Hepatitis B virus (HBV) to promote viral persistence (Kong et al., 2023), yet our recent work has revealed its protective role in mucosal immunity, where it defends against enteric bacterial infection by activating the IL-17F/β-defensin axis in colonic epithelium (Zhang et al., 2024). However, whether JMJD2D regulates IL-12b/IL-12p70-dependent immune responses during host defense against intracellular bacterial pathogens such as *L. monocytogenes* remains unknown.

In this study, we found that JMJD2D knockout (JMJD2D^-/-^) mice exhibited increased susceptibility to *L. monocytogenes* infection. Mechanistically, JMJD2D deficiency decreased the expression of NOD2, thereby affecting the ability of macrophages to recognize *L. monocytogenes*. JMJD2D also promoted IL-12b transcription by facilitating c-Rel and STAT1 activity and contributed to the reduction of repressive H3K9me3 enrichment at the IL-12b promoter. This regulation was associated with impaired IL-12p70 production and macrophage antimicrobial activity in JMJD2D-deficient cells. Additionally, JMJD2D participates in an IL-12p70-IFNγ regulatory circuit, in which IL-12p70 promotes IFNγ production by CD8^+^ T cells, whereas IFNγ enhances IL-12b expression in macrophages through the IFNGR2-JAK2-STAT1 pathway. Collectively, these findings indicate that JMJD2D contributes to host defense against *L. monocytogenes* infection, at least in part, by coordinating IL-12b expression, IL-12p70 production, and macrophage antimicrobial responses.

## 2 Materials and methods

### 2.1 Mice

Six- to eight-week-old male JMJD2D^-/-^ mice and wild-type (WT) littermate controls on a C57BL/6 background were used in this study. The JMJD2D^-/-^ mice were generated as previously described (Peng et al., 2019). The animal experimental design was approved by the Ethics Committee of Xiamen University (approval number: XMULAC20190042). All animal experiments were approved by the Xiamen University Laboratory Animal Center, Xiamen, Fujian, China.

### 2.2 Cell culture

HEK293T cells were maintained in DMEM medium (PM150210, Procell) supplemented with 10% FBS (Cat# C2810, VivaCell) and 100 U/mL penicillin-streptomycin. Bone marrow-derived macrophages (BMDMs) were isolated from mice according to the previous description and cultured in the medium containing 20 ng/mL M-CSF1 (RP01216, Abclonal) for 7 days (Chin et al., 2002). Peritoneal macrophages (PMs) were harvested 3 days after intraperitoneal injection of 4% thioglycollate broth (Cat# T9032, Sigma). BMDMs and PMs were cultured in RPMI 1640 medium (Cat# L210KJ, BasalMedia) supplemented with 10% FBS and 100 U/L penicillin-streptomycin. For STAT1 activation, BMDMs were stimulated with 20 ng/mL recombinant mouse IFNγ (Cat# RP01070, Abclonal) for 2 h for western blot analysis and for 6 hours for RT-qPCR analysis.

CD8^+^ T cells were isolated from C57BL/6 mice following an established protocol (Hong et al., 2024). Briefly, spleens were gently homogenized, passed through a 70 μm cell strainer, and subjected to red blood cell lysis. CD8^+^ T cells were then positively selected using anti-mouse CD8 microbeads (Cat# 130-117-044, Miltenyi). Cells were activated in RPMI-1640 complete medium supplemented with 10 ng/mL recombinant mouse IL-2 and 50 μM 2-mercaptoethanol. For the IFNγ activation experiment, CD8^+^ T cells were treated with 20 ng/mL recombinant mouse IL-12p70 (Cat# RP01696, Abclonal) for 24 h prior to flow cytometry. All cells were maintained in an incubator with 5% CO_2_ at 37°C.

### 2.3 Bacterial infection

*L. monocytogenes* strain 10403S was cultured in brain heart infusion (BHI) broth at 37°C with shaking (220 rpm) for 12 h or overnight. For *in vivo L. monocytogenes* infection, the mice were intraperitoneally injected with 2.5×10^6^ colony-forming units (CFU) of bacteria at the beginning of experiment. The intraperitoneal infection route was selected based on previous studies demonstrating its suitability for investigating host immune responses against *L. monocytogenes* infection (Carrero et al., 2006; Cassidy et al., 2020, 2022).

For *in vitro* infections, bacterial cultures were harvested by centrifugation and washed twice with PBS to remove the BHI medium. BMDMs or PMs were treated with heat-killed *L. monocytogenes* (MOI = 20) for the indicated time periods. For early signaling studies, cells were analyzed at 0, 30, 60, 120, and 240 min, whereas ChIP assays were performed at 2 h after stimulation. Cells were then washed twice with PBS and either lysed or processed for subsequent assays (Geng et al., 2021). In contrast, live *L. monocytogenes* was used for intracellular bacterial killing assays and *in vivo* infection experiments.

### 2.4 Bacterial burden in the organs

Three days post-infection, the spleen, lung, liver, cecum and feces of mice were collected. The tissue was weighed and placed in sterile PBS containing 1% TritonX-100 and ground at 40 Hz for 5 min on a tissue grinder at room temperature. The homogenate was blow-mixed evenly, and then diluted in a gradient with sterile PBS. The tissue homogenate suspension was spread onto BHI solid medium and incubated in a 37°C constant temperature incubator for 36 h. The bacteria on the medium were counted. Randomly selected single colonies for PCR detection, PCR primers: p60, Forward 5’-GCGGTAACAGCATTTGCTGCTCCAACAATC-3’, Reverse 5’-GCCATTGTCTTGCGCGTTAATCATTTGAC-3’.

### 2.5 Bacteria-killing assay

For the *in vitro* bacterial killing assay, fresh overnight cultures of *L. monocytogenes* were suspended in PBS and opsonized with fresh mouse serum. PMs pretreated with IFNγ (20 ng/mL) for 24 h were incubated with *L. monocytogenes* (MOI = 20) for 0, 2, 4, 6, 8 h at 37°C with intermittent shaking. After infection, cells were washed three times with sterile PBS to remove extracellular bacteria and subsequently incubated in complete medium containing gentamicin (100 μg/mL) for the indicated time points to eliminate any remaining extracellular bacteria. Cells were then lysed with 0.1% Triton X-100, serially diluted, and plated onto BHI agar plates. The CFU were counted after overnight incubation at 37°C and were used to quantify intracellular surviving bacteria.

For IL-12p70 rescue experiments, PMs were pretreated with recombinant mouse IL-12p70 (1 ng/mL) for 12 h prior to *L. monocytogenes* infection, and intracellular bacterial survival was assessed using the same gentamicin protection assay.

### 2.6 Real-time quantitative PCR (RT-qPCR)

Total RNA extraction was performed using RNAex Pro Reagent (Cat# AG21101, Accurate biology), then cDNA was obtained using the cDNA reverse transcription kit (Cat# G590, Applied Biological Materials). Real-time PCRs were performed using qPCR Master Mix (Cat# RK21203, Abclonal). Actin mRNA levels were used as the controls for normalization.

The sequences of RT-qPCR primers were listed as follows:

m-Actin, Forward 5’-ACTATTGGCAACGAGCGGTTCC-3’,
  Reverse 5’-GGCATAGAGGTCTTTACGGATGTCA-3’;
m-STAT1, Forward 5’-TCACAGTGGTTCGAGCTTCAG-3’,
  Reverse 5’-GCAAACGAGACATCATAGGCA-3’;
m-c-Rel, Forward 5’-AGAGGGGAATGCGGTTTAGAT-3’,
  Reverse 5’-TTCTGGTCCAAATTCTGCTTCAT-3’;
m-IL-6, Forward 5’-GACAAAGCCAGAGTCCTTCAGA-3’,
  Reverse 5’-TGTGACTCCAGCTTATCTCTTGG-3’;
m-IL-12b, Forward 5’-CGCCACACAAATGGATGCAA-3’,
  Reverse 5’-TGTGTCCTGAGGTAGCCGTA-3’;
m-iNOS, Forward 5’-GTTCTCAGCCCAACAATACAAGA-3’,
  Reverse 5’-GTGGACGGGTCGATGTCAC-3’;
m-TNFα, Forward 5’-ACGTGGAACTGGCAGAAGAG-3’,
  Reverse 5’-GGTCTGGGCCATAGAACTGA-3’;
m-IFNGR2, Forward 5’-TCCTCGCCAGACTCGTTTTC-3’;
  Reverse 5’-GTCTTGGGTCATTGCTGGAAG-3’.

### 2.7 Western blot

Cells or tissue samples were lysed in RIPA buffer supplemented with protease and phosphatase inhibitors. The protein concentration of the lysates was determined by the BCA quantitative method. Equal quality proteins were separated by SDS-PAGE and transferred to a PVDF membrane. After blocking with 5% skimmed milk for 1 h at room temperature, the membranes were incubated overnight at 4°C with the indicated primary antibodies. The following day, membranes were washed with TBST and incubated with appropriate horseradish peroxidase (HRP)-conjugated secondary antibodies for 1 h at room temperature. The signal was detected using ECL blotting substrates (Cat# RM00021P, Abclonal) and captured by a chemiluminescence imager (Tanon, China). The primary antibodies used in western blot were list as follows: anti-JMJD2D (Cat# ab289667, Abcam), anti-c-Rel (Cat# sc-71, Santa Cruz), anti-NOD2 (Cat# sc-56168, Santa Cruz), anti-STAT1 (Cat# 9172, CST), anti-p-STAT1 (Cat# 9167, CST), anti-JAK2 (Cat# 3230, CST), anti-p-JAK2 (Cat# 3776, CST), anti-p65 (Cat# 8242, CST), anti-p-p65 (Cat# 3033, CST), anti-β-actin (Cat# AC026, Abclonal), anti-HA-tag (Cat# AE105, Abclonal), anti-Flag-tag (Cat# AE092, Abclonal), anti-Myc-tag (Cat# AE070, Abclonal).

### 2.8 IL-12p70 and NO measurements

IL-12p70 in culture supernatants was measured by ELISA (Cat# MM-0174M1, MEIMIAN) according to the manufacturer’s instructions. NO production was assessed by measuring nitrite accumulation in culture supernatants using the Griess reagent (Cat# A012-1-2, Nanjing Jiancheng Bioengineering Institute).

### 2.9 SiRNA transfection

The siRNA targeting mouse *Stat1*, *c-Rel* and a non-targeting negative control were purchased from Sangon Biotech (Shanghai, China). PMs were transfected with the respective siRNAs using Jinbaoluo-siRNA Plus Transfection Reagent according to the manufacturer’s instructions.

### 2.10 Lentivirus infections

The coding sequence (CDS) of WT JMJD2D and its mutant were cloned into the pLV3-GFP-Puro lentiviral expression vector. For lentivirus production, HEK293T cells were co-transfected with the lentiviral transfer plasmid and the packaging plasmids (PMDL, VSVG and REV, at a mass ratio of 5:3:2). The virus-containing supernatant was collected 48 hours post-transfection, and either used immediately or aliquoted and stored at -80°C. PMs were infected with the harvested lentivirus for 24 hours. After 48 h of infection, the PMs were stimulated with *L. monocytogenes* (MOI = 20) for 6 h. Subsequently, total RNA was extracted from the cells for RT-qPCR analysis.

### 2.11 Luciferase reporter assay

IL-12b promoter reporter was constructed in PGL-3 basic vector for transfection. JMJD2D, STAT1 and c-Rel expression plasmids were constructed in pCMV5 vector. HEK293T cells were co-transfected with indicated plasmids and Renilla luciferase plasmid. The cells were collected 24 h after transfection. Luciferase activity was determined using the luciferase reporter assay system (Promega, Madison, US) as described previously (Peng et al., 2019).

### 2.12 Co-immunoprecipitation (Co-IP)

HEK293T cells were co-transfected with plasmids encoding the target proteins fused to different tags. At 24 h post-transfection, cells were lysed on ice using lysis buffer supplemented with protease and phosphatase inhibitor cocktails. For each sample, 5% of the total cell lysate was saved as input control. The remaining lysate was pre-cleared by incubation with Protein A/G Plus Agarose beads at 4°C for 1 hour with gentle rotation. Wash the agarose pellet three times with lysis buffer and once with washing buffer, and the samples were co-incubated overnight with the specified antibody and Protein A/G Plus Agarose at 4°C. The co-precipitates were isolated from the agarose magnetic beads, separated by SDS-PAGE, and analyzed by western blotting using antibodies against the relevant tags or proteins.

### 2.13 Chromatin immunoprecipitation (ChIP)

PMs isolated from JMJD2D^+/+^ and JMJD2D^-/-^ mice were treated with heat-killed *L. monocytogenes* (MOI = 20) for 2 h, respectively. Cells were then fixed with 1% formaldehyde for 10 minutes at room temperature to cross-link protein-DNA complexes. Chromatin was sheared by sonication to an average fragment size of 200–500 bp. Immunoprecipitation was performed using antibodies against H3K9me3, c-Rel, p-STAT1, and normal IgG (as a negative control). Protein A/G magnetic beads were used to capture the antibody-chromatin complexes. After extensive washing, the cross-links were reversed, and the co-precipitated DNA was purified. The enrichment of specific DNA fragments at the IL-12b promoter region was quantified by quantitative PCR using the following primers: Forward 5’-GGGGAGGGAGGAACTTCTTA-3’, Reverse 5’-TGATGGAAACCCAAAGTAGAAACTGAC-3’.

### 2.14 Flow cytometer

Splenocytes were isolated by gently grinding the spleen in RPMI-1640 medium and passing the homogenate through a 70 μm cell strainer. Cells were incubated with the corresponding fluorophore conjugated antibody for 30 min in the dark at 4°C. For intracellular cytokine staining, single-cell suspensions were stimulated for 6 h at 37°C in complete medium containing 50 ng/mL phorbol 12-myristate 13-acetate (PMA; Cat# P1585, Sigma), 1 μg/mL ionomycin (Cat# I139530, Aladdin), and GolgiPlug (Cat# 51-2301kz, BD Biosciences). After staining the cell surface with antibodies, cells were fixed and permeabilized using a commercial fixation/permeabilization solution (Cat# 51-2090kz, BD Biosciences) for 30 min at 4°C. After washing, intracellular cytokines were stained with the corresponding antibodies for 30 min at 4°C in the dark. Stained cells were analyzed using a BD Fortessa X-20 or CytoFLEX LX flow cytometer. Data were processed and analyzed with FlowJo X software.

The fluorescence conjugated antibodies used in this study were listed as follows: APC/Cy7 CD45 (Cat# 103116, BioLegend), Fixable Viability Dye eFluor™ 506 (Cat# 65-0866-18, Invitrogen), PE Gr-1 (Cat# 108408, BioLegend), PE-Cyanine7 F4/80 (Cat# 123114, BioLegend), PerCP/Cy5.5 CD11b (Cat# 101228, BioLegend), BV421 CD86 (Cat# 105032, BioLegend), FITC MHCII (Cat# 115006, BioLegend), BV785 TCRβ (Cat# 109249, BioLegend), PerCP/Cy5.5 CD4 (Cat# A14785, Invitrogen), APC/Cy7 CD8 (Cat# A15386, BioLegend), APC IFNγ (Cat# 505809, BioLegend), APC IL-12b (Cat# 554480, BD Pharmingen).

### 2.15 Histological analysis

Colon and spleen tissues were fixed in 10% neutral buffered formalin for 18 h at room temperature. After fixation, tissues were dehydrated through a graded ethanol series, cleared in xylene, and embedded in paraffin wax. Sections (5 μm thick) were cut using a microtome, mounted on glass slides, and dried. For histological examination, sections were deparaffinized, rehydrated, and stained with hematoxylin and eosin (H&E) according to standard protocols. Stained sections were imaged using a Leica DM4B light microscope equipped with a digital camera.

### 2.16 Statistical analysis

Statistical analyses were performed using GraphPad Prism (GraphPad Software, Version 8). Quantitative data were normally distributed and are presented as the mean ± SEM. Survival curves were analyzed using the log-rank (Mantel-Cox) test. For comparisons between two groups, an unpaired two-tailed Student’s t-test was used. For experiments involving two independent factors, ordinary two-way ANOVA was performed, followed by Šídák’s multiple-comparisons test or pre-planned Fisher’s LSD comparisons. Pre-planned comparisons were defined *a priori* based on the experimental design. For one-way comparisons involving more than two groups, one-way ANOVA followed by Tukey’s multiple-comparisons test was used. The specific statistical test used for each experiment is indicated in the corresponding figure legend. A two-sided *p* < 0.05 was considered statistically significant.

## 3 Results

### 3.1 JMJD2D deficiency in mice exacerbates susceptibility to *L. monocytogenes* infection

To explore a potential role of JMJD2D in *L. monocytogenes* infection, we first examined JMJD2D expression in response to *L. monocytogenes* infection. As shown in **Supplementary Figures 1A, B**, JMJD2D expression was markedly increased in the BMDMs upon *L. monocytogenes* stimulation, suggesting its potential involvement in the host antibacterial response. Given that NF-κB signaling is activated during *L. monocytogenes* infection (Soria-Castro et al., 2021) and that JMJD2D can be induced by TNFα through NF-κB in other inflammatory contexts (Zhuo et al., 2020), we next examined p65 phosphorylation. Indeed, p-p65 levels were increased in BMDMs upon *L. monocytogenes* treatment (**Supplementary Figures 1A, C**), raising the possibility that JMJD2D may be part of the innate immune response to *L. monocytogenes*.

To determine whether JMJD2D influences host susceptibility *in vivo*, we established a systemic murine infection model by intraperitoneally injecting WT and JMJD2D^-/-^ mice with 2.5×10^6^ CFU of *L. monocytogenes*, and disease progression was monitored for up to 8 days. To confirm the identity of the recovered bacteria, colonies from tissue homogenates were randomly selected and subjected to PCR targeting the *L. monocytogenes*-specific *p60* gene. The consistent detection of the expected 1300 bp amplicon verified that the quantified colonies were indeed *L. monocytogenes* (**Supplementary Figure 1D**). Following infection, JMJD2D^-/-^ mice exhibited significantly greater body weight loss from days 2 to 7 compared to WT controls, as shown by the percentage change from initial body weight (**Figure 1A**), with consistent individual body weight trajectories across animals (**Supplementary Figure 1E**). Moreover, analysis of absolute body weight showed a consistent trend: WT mice recovered after an initial decline, whereas JMJD2D^-/-^ mice exhibited a sustained decrease throughout the observation period, although the difference did not reach statistical significance (**Supplementary Figure 1F**), supporting that JMJD2D deficiency exacerbates disease severity upon *L. monocytogenes* infection. Strikingly, 75% of JMJD2D^-/-^ mice (6/8) succumbed to the infection by day 8, whereas all WT mice survived throughout the observation period (**Figure 1B**). Consistent with this heightened mortality, JMJD2D**^-/-^** mice harbored significantly higher bacterial burdens in the cecum, spleen, lung, and liver on day 3 post-infection (**Figure 1C**), indicating that JMJD2D^-/-^ mice exhibit more severe systemic dissemination of *L. monocytogenes* compared to WT mice. Given that the colon and spleen are primary target organs in listeriosis (Jones et al., 2015), we examined the pathological damage in these tissues after *L. monocytogenes* injection. The colon length was significantly shortened in infected JMJD2D^-/-^ mice compared to WT controls, whereas no such difference was observed under naive conditions (**Figures 1D, E**). Histological analysis by H&E staining showed that prior to infection, the colonic architecture of JMJD2D^-/-^ mice was comparable to that of WT mice, ruling out spontaneous colonic pathology due to JMJD2D deficiency (**Figure 1F**). On day 3 post-infection, both WT and JMJD2D^-/-^ mice exhibited pathological changes in the colon and spleen, but the degree of tissue damage was more severe in JMJD2D^-/-^ mice (**Figures 1F, G**). Infected JMJD2D^-/-^ mice exhibited severe damage to the colonic mucosa, with edema of the submucosa, a decrease in epithelial integrity, and infiltration of inflammatory cells in the muscular layer (**Figure 1F**). Similarly, infected JMJD2D^-/-^ spleens exhibited destruction of white pulp structures and loss of distinct marginal zones (**Figure 1G**). Taken together, these results suggest that JMJD2D plays a protective role in host defense against *L. monocytogenes* infection.

**Figure 1:**
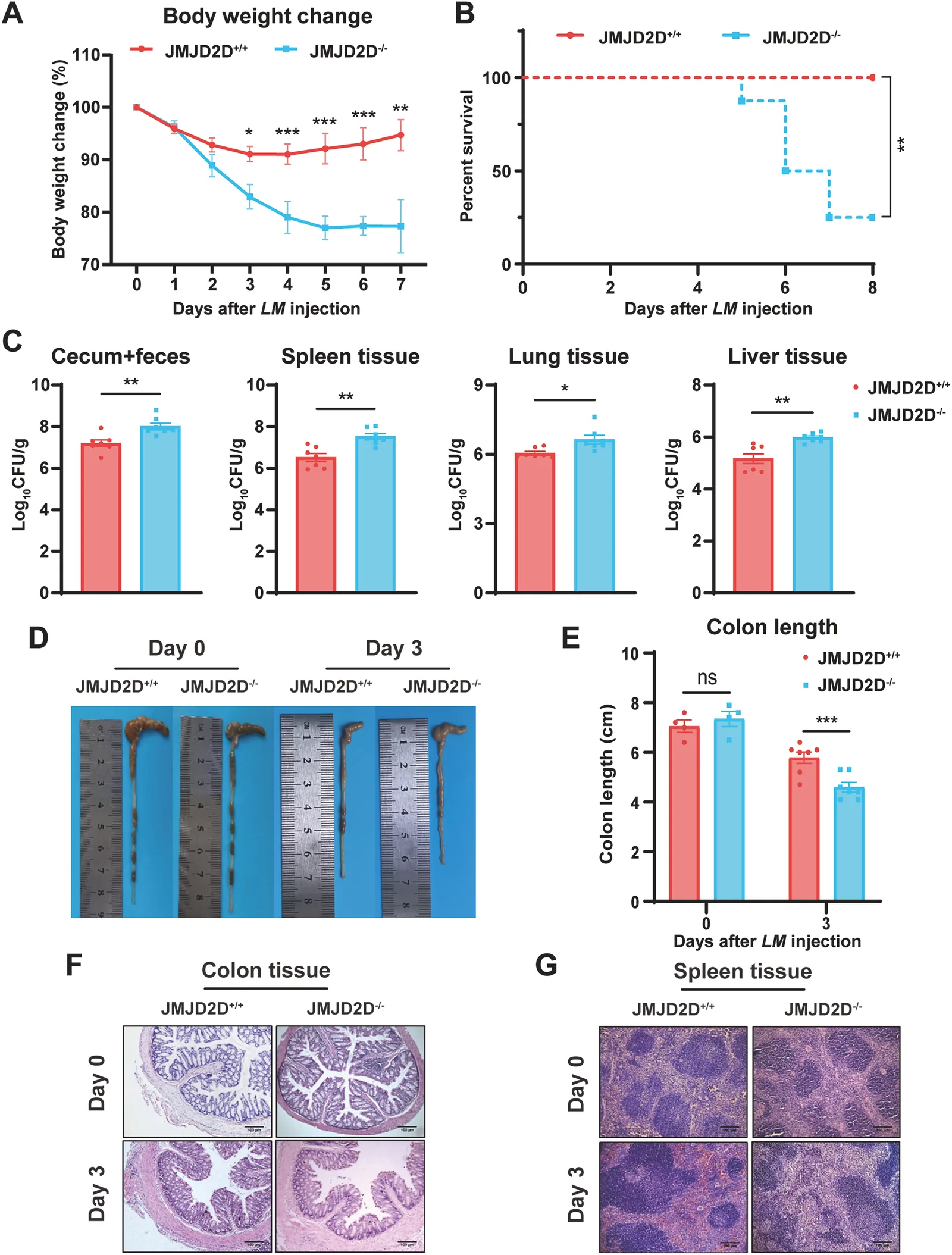
JMJD2D deficiency exacerbates susceptibility to *L. monocytogenes* infection. **(A)** JMJD2D^-/-^ mice exhibited greater body weight loss compared to WT controls after infection (*n* = 8 per group). **(B)** Survival curves of WT and JMJD2D^-/-^ mice after *L. monocytogenes* infection, monitored for 8 days (*n* = 8 per group). **(C)** Bacterial burdens were higher in the cecum+feces, spleen, lung, and liver of JMJD2D^-/-^ mice at day 3 post-infection (*n* = 7). **(D, E)** Infection led to a shortening of colon length in JMJD2D^-/-^ mice at day 3 post-infection (*n* = 7). **(F)** H&E staining revealed that infected JMJD2D^-/-^ mice showed severe mucosal damage, edema, and inflammatory infiltration. Scale bars, 100 µm. **(G)** H&E staining revealed that infected JMJD2D^-/-^ spleens displayed destruction of white pulp and loss of marginal zones. Scale bars, 100 µm. Data are shown as mean ± SEM. Statistical analysis: **(A)** two-way ANOVA followed by Šídák’s multiple-comparisons test; **(B)** log-rank (Mantel-Cox) test; **(C)** unpaired two-tailed Student’s t-test; **(E)** two-way ANOVA with pre-planned Fisher’s LSD comparisons. Results are representative of three independent experiments. ^ns^*p*>0.05; *p < 0.05; **p < 0.01; ***p < 0.001.

### 3.2 JMJD2D deficiency impairs bacterial clearance by suppressing M1 macrophage polarization and CD8^+^ T cell IFNγ responses

Effective host defense against *L. monocytogenes* requires coordinated innate and adaptive immune responses (Calame et al., 2016; D'Orazio, 2019). Macrophages and other myeloid cells constitute the first line of defense by recognizing and eliminating invading bacteria, thereby restricting early pathogen expansion (Hirayama et al., 2017). In parallel, antigen-specific T cells mediate the final clearance of intracellular bacteria through cytotoxic activity and the production of effector cytokines such as IFNγ (Bold and Ernst, 2012). To determine whether JMJD2D contributes to immune protection during *L. monocytogenes* infection, we first examined immune cell infiltration in the spleen 3 days after infection. Flow cytometry revealed that *L. monocytogenes* infection did not significantly alter the proportion of macrophages or CD4^+^ T cells in either genotype (**Figures 2A, B**; **Supplementary Figures 2A, B**). Although splenic CD8^+^ T cells showed a trend toward reduction following infection, neither the frequency nor the absolute number of CD8^+^ T cells was significantly altered in infected JMJD2D^-/-^ mice compared with WT controls (**Figure 2B****;** **Supplementary Figure 2C**). Similarly, the absolute numbers of splenic CD4^+^ T cells were comparable between genotypes following infection (**Supplementary Figure 2D**), with no apparent genotype-dependent difference in overall splenic T cell abundance. We next assessed the impact of JMJD2D on macrophage activation. Upon *L. monocytogenes* stimulation, the production of pro-inflammatory cytokines IL-6 and TNFα was markedly induced both *in vivo* and *in vitro*, whereas JMJD2D deficiency substantially blunted these increases (**Supplementary Figures 3A–F**). Consistent with an impaired innate response, the frequency of CD86^+^ M1 macrophages was significantly lower in infected JMJD2D^-/-^ mice (**Figures 2C, D**). Given the close interplay between macrophages and T cells, we further assessed adaptive immunity. The level of IFNγ produced by CD8^+^ T cells was elevated in infected WT mice but significantly reduced in JMJD2D^-/-^ mice (**Figures 2E, F**).

**Figure 2:**
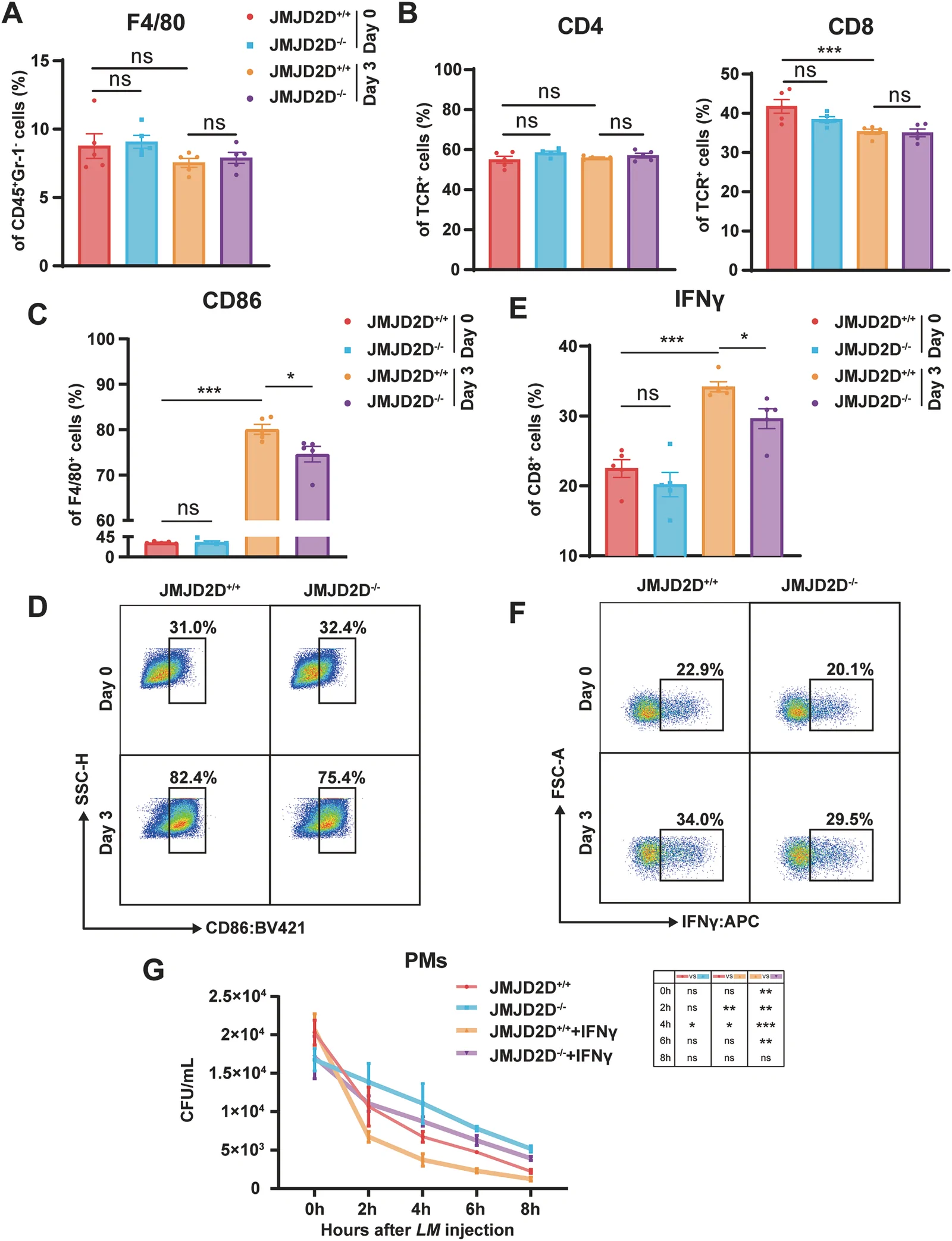
JMJD2D supports macrophage antimicrobial function and CD8^+^ T cell IFNγ responses during *L. monocytogenes* infection. **(A)** Flow cytometry showed that *L. monocytogenes* infection did not significantly alter the proportion of macrophages at day 3 post-infection (*n* = 5). **(B)** Flow cytometry showed that a reduction in the percentage of CD8^+^ T cells, but not CD4^+^ T cells, at day 3 post-infection (*n* = 5). **(C, D)** The frequency of CD86^+^ M1 macrophages was reduced in infected JMJD2D^-/-^ mice compared to infected WT controls (*n* = 5). **(E, F)** The production of IFNγ by CD8^+^ T cells was reduced in infected JMJD2D^-/-^ mice compared to infected WT controls (*n* = 5). **(G)** Intracellular bacterial killing was assessed using a gentamicin protection assay with live *L. monocytogenes*. JMJD2D^-/-^ PMs exhibited impaired intracellular bactericidal activity, resulting in higher intracellular bacterial CFU than WT cells, even after IFNγ stimulation (*n* = 3). Data are shown as mean ± SEM. Statistical analysis: **(A–C, E)** two-way ANOVA with pre-planned Fisher’s LSD comparisons; **(G)** two-way ANOVA followed by Šídák’s multiple-comparisons test. ^ns^*p*>0.05; *p < 0.05; **p < 0.01; ***p < 0.001.

To determine whether these defective effector responses resulted in diminished antibacterial activity, we measured classical antibacterial effector molecules. JMJD2D deficiency led to a significant decrease in inducible nitric oxide synthase (iNOS) mRNA and protein levels in infected macrophages (**Supplementary Figure 3G**). Consistently, NO production was also reduced in JMJD2D-deficient macrophages upon *L. monocytogenes* stimulation (**Supplementary Figure 3H**). Functionally, PMs from JMJD2D^-/-^ mice exhibited a markedly reduced capacity to kill intracellular *L. monocytogenes*, as evidenced by higher bacterial survival (**Figure 2G**). This impaired bactericidal activity correlated with deficient bacterial engulfment and clearance, even in the presence of IFNγ (**Figure 2G**). Together, these findings demonstrate that JMJD2D contributes to effective antibacterial immunity by promoting macrophage antimicrobial responses and supporting CD8^+^ T cell IFNγ production during *L. monocytogenes* infection.

### 3.3 JMJD2D promotes macrophage IL-12b expression and IL-12p70 production during *L. monocytogenes* infection

IL-12 is a pivotal cytokine in antimicrobial responses to intracellular pathogens, promoting macrophage activation and CD8^+^ T cell effector functions (Mansurov et al., 2020). Given the impaired macrophage function and IFNγ response observed in JMJD2D^-/-^ mice (**Figure 2**), we hypothesized that JMJD2D contributes to antimicrobial immunity by regulating the IL-12 axis. To test this, we first examined IL-12b expression *in vivo*. Flow cytometry analysis revealed that splenic macrophages from infected JMJD2D^-/-^ mice expressed lower levels of intracellular IL-12b than those from WT controls (**Figures 3A, B**). This reduction was further supported at the transcriptional level, as RT-qPCR showed that the robust upregulation of IL-12b mRNA in infected WT spleens was markedly attenuated in JMJD2D^-/-^ mice (**Figure 3C**). We next examined whether JMJD2D deficiency also affected IL-12b expression in macrophages *in vitro*. Following *L. monocytogenes* stimulation, both BMDMs and PMs from JMJD2D^-/-^ mice exhibited significantly lower IL-12b mRNA and intracellular IL-12b levels than their WT counterparts (**Figures 3D–F**).

**Figure 3:**
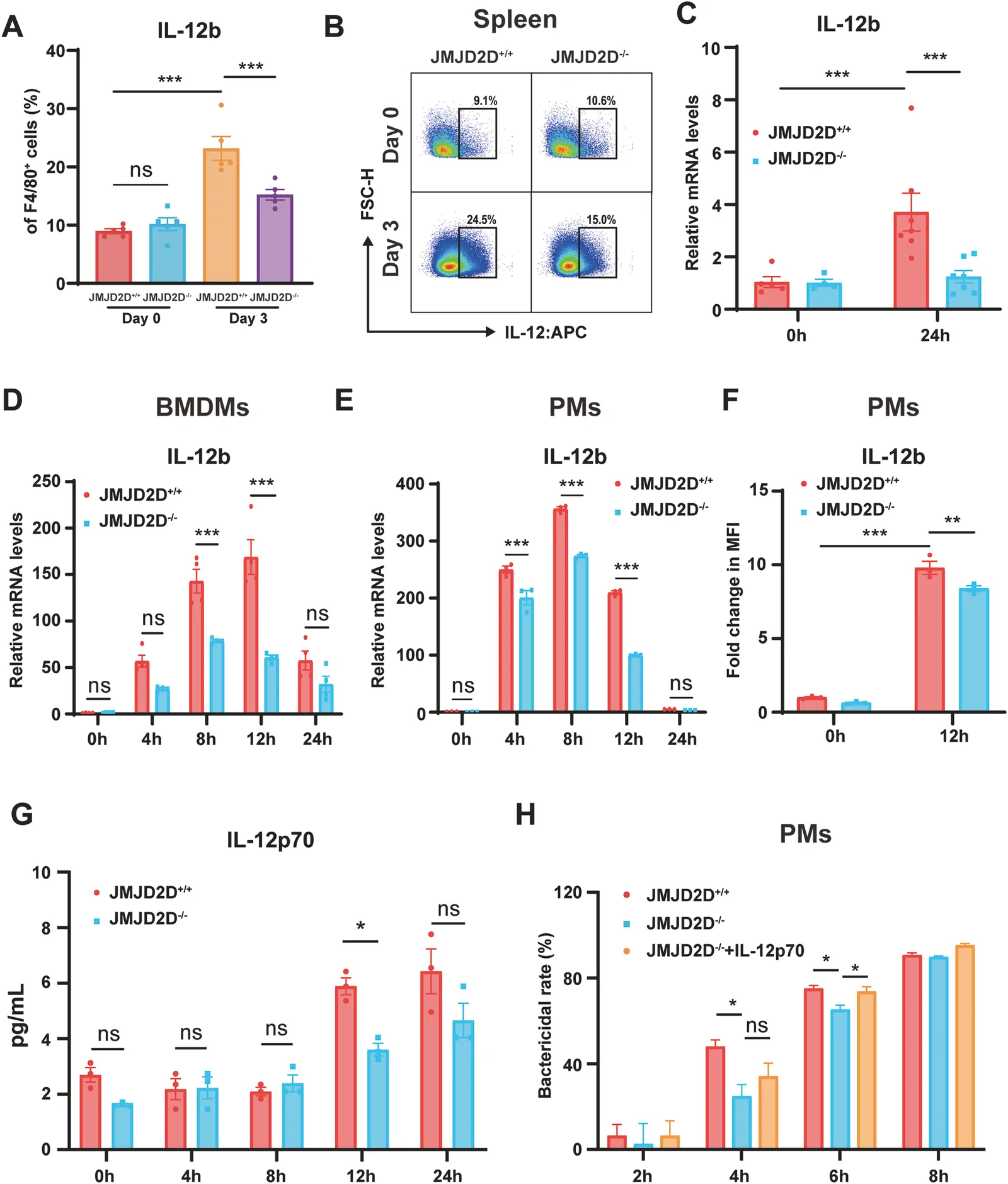
JMJD2D regulates IL-12b expression and IL-12p70 production during *L. monocytogenes* infection. **(A-B)** Splenic macrophages from infected JMJD2D^-/-^ mice expressed lower levels of IL-12b than those from WT mice (*n* = 5). **(C)** The infection-induced increase in splenic IL-12b mRNA was suppressed in JMJD2D^-/-^ mice (*n* = 7). **(D)** IL-12b mRNA induction by *L. monocytogenes* was reduced in JMJD2D^-/-^ BMDMs (*n* = 4). **(E)** IL-12b mRNA induction by *L. monocytogenes* was reduced in JMJD2D^-/-^ PMs (*n* = 3). **(F)** Intracellular IL-12b expression in infected BMDMs was lower in the absence of JMJD2D (*n* = 3). **(G)** JMJD2D deficiency reduced secreted IL-12p70 production at 12 h post *L. monocytogenes* stimulation, as measured by ELISA (*n* = 3). **(H)** IL-12p70 pretreatment partially rescues the impaired intracellular killing of live *L. monocytogenes* by JMJD2D-deficient macrophages, as assessed by a gentamicin protection assay (*n* = 3). Data are shown as mean ± SEM. Statistical analysis: **(A, C, F)** two-way ANOVA with pre-planned Fisher’s LSD comparisons; **(D, E, G)** two-way ANOVA followed by Šídák’s multiple-comparisons test; **(H)** one-way ANOVA followed by Tukey’s multiple-comparisons test. The experiments were repeated three times independently. ^ns^*p*>0.05; *p < 0.05; **p < 0.01; ***p < 0.001.

Because IL-12b is a component of the bioactive IL-12p70 heterodimer, we next examined whether the reduction in IL-12b expression was accompanied by impaired secretion of functional IL-12p70. ELISA analysis showed that *L. monocytogenes* stimulation induced IL-12p70 secretion in WT macrophages, whereas this induction was significantly impaired in JMJD2D-deficient macrophages (**Figure 3G**). To determine whether IL-12p70 restoration could rescue the impaired bactericidal activity of JMJD2D-deficient macrophages, we performed a rescue experiment. As shown in **Figures 3H**, IL-12p70 pretreatment partially but significantly restored the bactericidal activity of JMJD2D^-/-^ macrophages at 6 h post-infection (**Figure 3H**). Collectively, these data demonstrate that JMJD2D promotes IL-12b expression and IL-12p70 production in macrophages, supporting a functional contribution of IL-12p70 to the antimicrobial defects associated with JMJD2D deficiency.

### 3.4 JMJD2D cooperates with c-Rel to promote IL-12b expression in macrophages

During *L. monocytogenes* infection, macrophages recognize pathogen-associated molecular patterns (PAMPs) through pattern-recognition receptors (PRRs), including the intracellular sensor NOD2 (Girardin et al., 2003; Jeong et al., 2014). Consistent with its role in bacterial sensing and with previous reports showing that NOD2 expression is upregulated upon macrophage activation (Lee et al., 2012; Pang et al., 2026), NOD2 protein levels increased rapidly during the early phase of bacterial stimulation in both BMDMs (**Figures 4A, B**) and PMs (**Figures 4C, D**). We next asked whether JMJD2D deficiency affected this response. Indeed, JMJD2D deficiency attenuated the infection-induced increase in NOD2 protein level in macrophages (**Figures 4A–D**). Upon ligand recognition, NOD2 activates NF-κB signaling, which promotes the transcription of pro-inflammatory cytokines including IL-12b (Swain and Miryala, 2025). Because the NF-κB binding site within the IL-12b promoter preferentially recruits c-Rel in macrophages (Cuesta et al., 2025), we next examined whether JMJD2D deficiency affects c-Rel expression and whether c-Rel is required for IL-12b transcription. Western blot analysis showed that the early induction of c-Rel protein following *L. monocytogenes* stimulation was markedly impaired in JMJD2D^-/-^ macrophages (**Figures 4E–H**), in line with previous findings that c-Rel is induced upon macrophage activation (DeLaney et al., 2019). Moreover, siRNA-mediated knockdown of c-Rel in PMs significantly reduced IL-12b expression following infection (**Figure 4I**).

**Figure 4:**
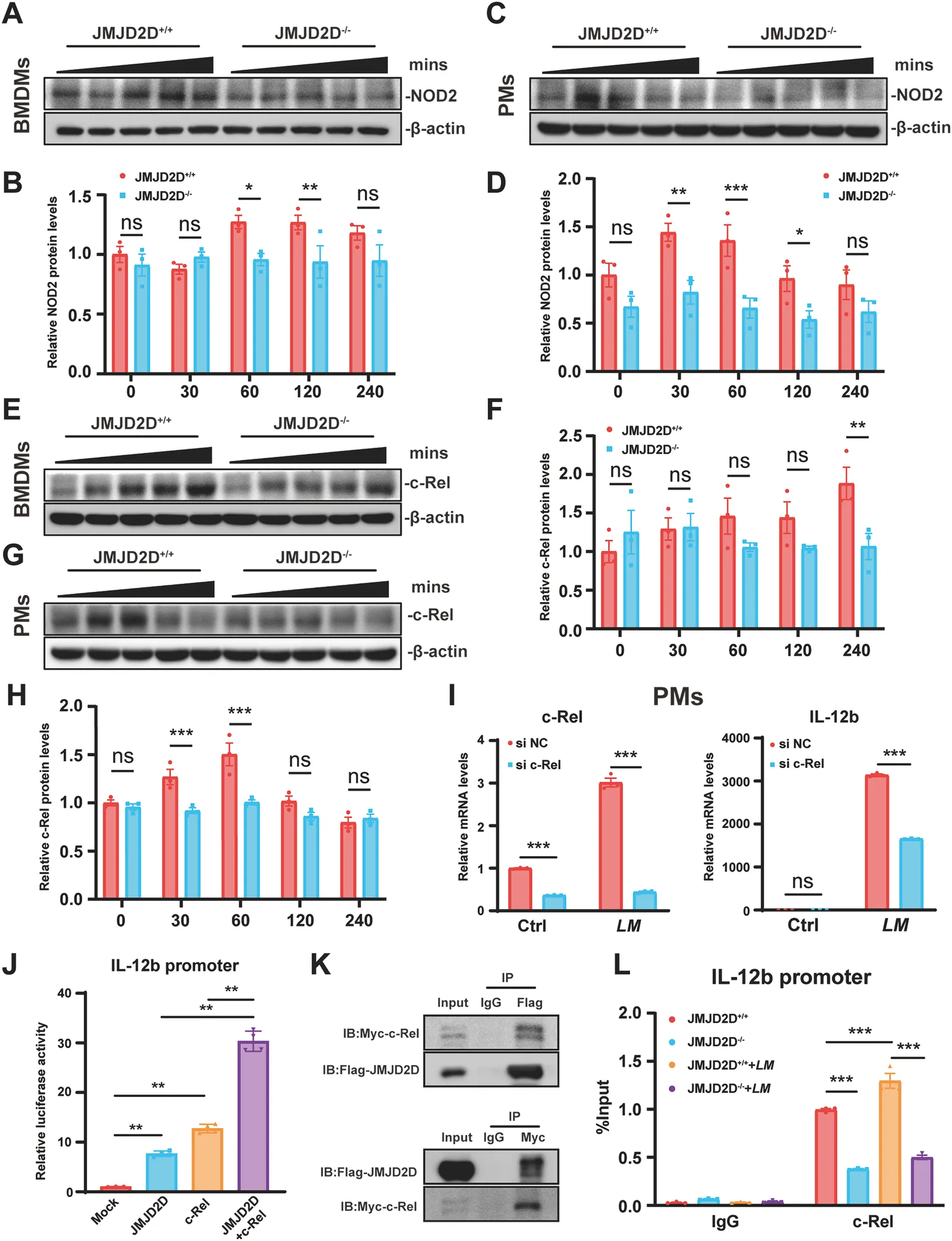
JMJD2D regulates IL-12b transcription through the NOD2-NF-κB/c-Rel pathway. **(A–D)** NOD2 protein expression was increased in both BMDMs **(A, B)** and PMs **(C, D)** upon *L. monocytogenes* stimulation (0, 30, 60, 120, and 240 min), and this induction was attenuated by JMJD2D deficiency (*n* = 3). **(E–H)** The increase in c-Rel protein level upon infection was inhibited in JMJD2D^-/-^ BMDMs **(E, F)** and PMs **(G, H)** following *L. monocytogenes* stimulation (0, 30, 60, 120, and 240 min) (*n* = 3). **(I)** Knockdown of c-Rel in WT PMs reduced infection-induced IL-12b mRNA expression (*n* = 3). **(J)** Luciferase reporter assay showed that JMJD2D and c-Rel synergistically activated the IL-12b promoter (*n* = 3-4). **(K)** Co-IP assay confirmed a physical interaction between JMJD2D and c-Rel. **(L)** ChIP assay revealed that JMJD2D deficiency reduced the recruitment of c-Rel to the IL-12b promoter after *L. monocytogenes* stimulation for 2 h (*n* = 3). Data are shown as mean ± SEM. Statistical analysis: **(B, D, F, H, I)** two-way ANOVA with pre-planned Fisher’s LSD comparisons; **(J)** one-way ANOVA followed by Tukey’s multiple-comparisons test; **(L)** two-way ANOVA followed by Šídák’s multiple-comparisons test. The experiments were repeated three times independently. ^ns^*p*>0.05; *p < 0.05; **p < 0.05; ***p < 0.001.

To further investigate the underlying mechanism, we performed luciferase reporter assays, which showed that JMJD2D cooperates with c-Rel to synergistically activate the IL-12b promoter (**Figure 4J**). This functional cooperation was further supported by a physical interaction between JMJD2D and c-Rel, as evidenced by Co-IP assays (**Figure 4K**). Given these findings, we next tested whether JMJD2D facilitates c-Rel recruitment to the IL-12b promoter. ChIP assays showed that *L. monocytogenes* infection increased c-Rel enrichment at the IL-12b promoter in WT macrophages at 2 h post-infection, but this recruitment was significantly diminished in JMJD2D-deficient cells (**Figure 4L**). Collectively, these results support a model in which JMJD2D acts as a transcriptional coactivator to facilitate c-Rel recruitment to the IL-12b promoter, thereby promoting IL-12b transcription during *L. monocytogenes* infection.

### 3.5 JMJD2D regulates IFNγ-induced IL-12b expression through the IFNGR2-JAK2-STAT1 axis

Building on these findings, we next investigated the reciprocal regulation between macrophage IL-12p70 production and CD8^+^ T cell IFNγ responses. To determine whether IL-12p70 directly promotes CD8^+^ T cell effector function, we treated CD8^+^ T cells with recombinant IL-12p70 (25 or 50 ng/mL) and observed a dose-dependent increase in IFNγ production (**Supplementary Figures 4A, B**). We next asked whether macrophage-derived signals could similarly influence this response. Conditioned medium (CM) from WT or JMJD2D^-/-^ PMs was collected and used to stimulate CD8^+^ T cells. Compared with CM from WT PMs, CM from JMJD2D^-/-^ PMs markedly reduced both the proportion and mean fluorescence intensity (MFI) of IFNγ^+^CD8^+^ T cells, regardless of prior *L. monocytogenes* stimulation (**Figures 5A–C**; **Supplementary Figure 4C**), indicating that JMJD2D deficiency in macrophages diminishes their capacity to promote CD8^+^ T cell IFNγ production. We next examined whether IFNγ could reciprocally enhance macrophage IL-12b expression, providing a potential basis for reciprocal regulation between macrophages and CD8^+^ T cells. Stimulation of BMDMs with recombinant IFNγ robustly upregulated IL-12b mRNA, but this induction was significantly attenuated in JMJD2D^-/-^ cells (**Figure 5D**), indicating that JMJD2D contributes to the ability of macrophages to respond to IFNγ and enhance IL-12b expression.

**Figure 5:**
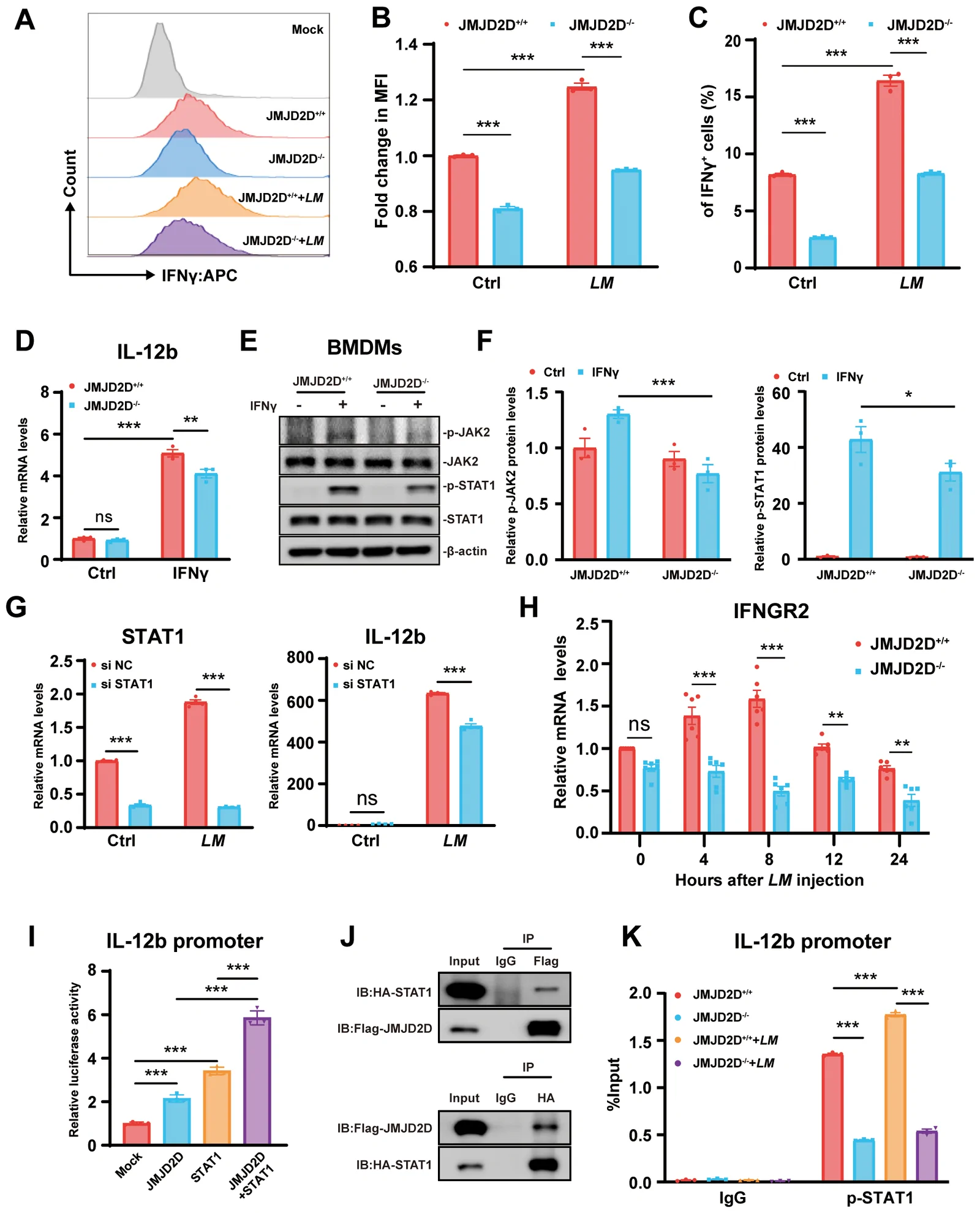
JMJD2D facilitates IFNγ-dependent regulation of IL-12b expression through the IFNGR2-JAK2-STAT1 pathway. **(A–C)** CM from JMJD2D^-/-^ PMs was less effective in stimulating both the MFI **(A, B)** and frequency **(C)** of IFNγ production in CD8^+^ T cells (*n* = 3). **(D)** IFNγ-induced IL-12b mRNA upregulation in BMDMs was inhibited by JMJD2D deficiency (*n* = 3). **(E, F)** IFNγ-triggered phosphorylation of JAK2 and STAT1 was attenuated in JMJD2D^-/-^ BMDMs (*n* = 3). **(G)** STAT1 knockdown in WT PMs suppressed infection-induced IL-12b mRNA expression (*n* = 4). **(H)** JMJD2D deficiency led to decreased IFNGR2 mRNA levels in BMDMs (*n* = 6). **(I)** Luciferase assay showed that JMJD2D and STAT1 cooperatively activate the IL-12b promoter. **(J)** Co-IP confirmed an interaction between JMJD2D and STAT1. **(K)** ChIP assay showed that JMJD2D deficiency reduced STAT1 enrichment at the IL-12b promoter after *L. monocytogenes* stimulation for 2 h (*n* = 3). Data are shown as mean ± SEM. Statistical analysis: **(B–D, F, G)** two-way ANOVA with pre-planned Fisher’s LSD comparisons; **(H, K)** two-way ANOVA followed by Šídák’s multiple-comparisons test; **(I)** one-way ANOVA followed by Tukey’s multiple-comparisons test. The experiments were repeated three times independently. ^ns^*p*>0.05; *p < 0.05; **p < 0.01; ***p < 0.001.

Canonical IFNγ signaling proceeds through the JAK-STAT pathway in various cell types, including macrophages (Ivashkiv, 2018). To define the molecular basis of the IFNγ-dependent regulation observed above, we examined this pathway in JMJD2D-deficient macrophages. JMJD2D deficiency blunted the phosphorylation of STAT1 in both BMDMs and PMs after *L. monocytogenes* infection (**Supplementary Figures 4D, E**). Consistent with this finding, IFNγ stimulation rapidly induced phosphorylation of JAK2 and STAT1 in WT BMDMs, whereas both responses were significantly reduced in JMJD2D^-/-^ cells (**Figures 5E, F**). The functional necessity of STAT1 was further confirmed by siRNA knockdown, which markedly suppressed infection-induced IL-12b expression in PMs (**Figure 5G**). Given that IFNγ receptor is responsible for the initiation of JAK-STAT signaling (Han et al., 2023), we hypothesized that JMJD2D might regulate the expression of its receptor. Indeed, JMJD2D deficiency significantly downregulated the expression of the receptor subunit IFNGR2 both in infected BMDMs *in vitro* and in the spleens of infected mice *in vivo* (**Figure 5H**; **Supplementary Figure 4F**), suggesting that impaired IFNγ signaling in JMJD2D-deficient macrophages may arise, at least in part, from reduced IFNGR2 expression.

Given the upstream role of c-Rel in IL-12b transcription identified earlier, we next explored the relationship between c-Rel and STAT1. Knockdown experiments revealed a unidirectional regulatory relationship, in which silencing c-Rel markedly reduced STAT1 expression regardless of *L. monocytogenes* stimulation, whereas STAT1 knockdown had no significant impact on c-Rel levels (**Supplementary Figure 4G**). This places c-Rel upstream of STAT1 in the regulatory hierarchy and suggests that the JMJD2D-c-Rel pathway may contribute to amplifying STAT1-dependent IL-12b transcription.

To determine whether JMJD2D directly cooperates with STAT1 at the IL-12b promoter, dual-luciferase reporter assays were performed. JMJD2D and STAT1 independently enhanced the IL-12b promoter activity, and their co-expression produced a synergistic effect (**Figure 5I**). Co-IP confirmed a physical interaction between JMJD2D and STAT1 (**Figure 5J**). Consistently, ChIP assays showed that *L. monocytogenes* infection increased p-STAT1 enrichment at the IL-12b promoter in WT macrophages, whereas this enrichment was significantly reduced in JMJD2D^-/-^ cells (**Figure 5K**). These results support a role for JMJD2D as a transcriptional cofactor that facilitates STAT1 recruitment to the IL-12b promoter.

Collectively, these findings establish that JMJD2D promotes IFNγ-induced IL-12b expression in macrophages through the IFNGR2-JAK2-STAT1 pathway, positioning JMJD2D as a critical regulator of macrophage responsiveness to IFNγ.

### 3.6 Enzymatic activity of JMJD2D contributes to IL-12b expression and macrophage inflammatory responses during *L. monocytogenes* infection

Having shown that JMJD2D cooperates with c-Rel and STAT1 in regulating IL-12b promoter activity, we sought to determine whether its canonical histone demethylase activity contributes to this immunoregulatory function. WT PMs were transduced with lentiviral vectors expressing either JMJD2D-WT or the catalytically inactive mutant JMJD2D-S200M. Successful overexpression of both constructs was confirmed at the mRNA level (**Figure 6A**). We first examined the effect of JMJD2D catalytic activity on IL-12b expression. Overexpression of JMJD2D-WT markedly enhanced IL-12b expression following *L. monocytogenes* infection, whereas the JMJD2D-S200M mutant produced only a modest increase (**Figure 6B**), indicating that the catalytic activity of JMJD2D contributes substantially to the full induction of IL-12b. We next investigated whether this catalytic requirement extended to other macrophage inflammatory responses. JMJD2D-WT overexpression robustly increased the expression of the pro-inflammatory cytokine IL-6 and the bactericidal mediator iNOS, whereas JMJD2D-S200M failed to induce iNOS and elicited a significantly weaker increase in IL-6 (**Figures 6C, D**).

**Figure 6:**
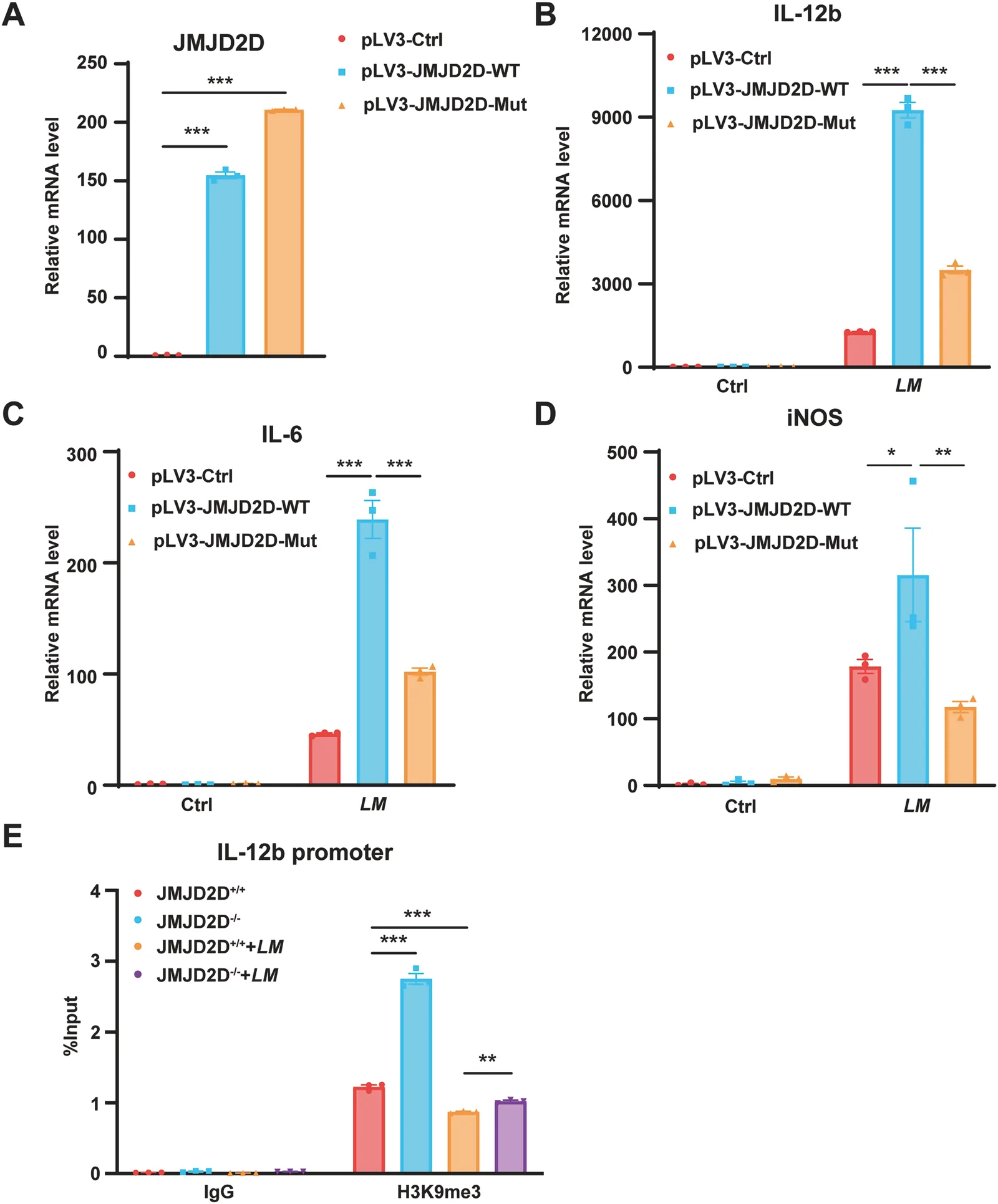
JMJD2D catalytic activity contributes to IL-12b expression and acrophage inflammatory responses during *L. monocytogenes* infection. **(A)** Successful overexpression of JMJD2D-WT and JMJD2D-S200M in WT PMs (*n* = 3). **(B)** JMJD2D-WT overexpression strongly enhanced IL-12b expression following *L. monocytogenes* infection, while the JMJD2D-S200M mutant induced a modest increase. **(C, D)** JMJD2D-WT overexpression potently increased IL-6 **(C)** and iNOS **(D)** mRNA levels upon *L. monocytogenes* infection, while the JMJD2D-S200M mutant produced a weaker IL-6 induction and failed to induce iNOS (*n* = 3). **(E)** The reduction in H3K9me3 enrichment at the IL-12b promoter induced by 2 h of *L. monocytogenes* stimulation in WT PMs was abolished in JMJD2D^-/-^ PMs (*n* = 3). Data are shown as mean ± SEM. Statistical analysis: **(A)** one-way ANOVA followed by Tukey’s multiple-comparisons test; **(B–E)** two-way ANOVA followed by Šídák’s multiple-comparisons test. The experiments were repeated three times independently. *p < 0.05; **p < 0.01; ***p < 0.001.

To explore the epigenetic basis for this requirement, we examined H3K9me3 enrichment at the IL-12b promoter. In WT PMs, *L. monocytogenes* infection significantly reduced H3K9me3 enrichment at the IL-12b promoter, whereas this reduction was partially prevented in JMJD2D^-/-^ macrophages, with H3K9me3 levels intermediate between those of uninfected WT and infected WT cells (**Figure 6E**). These findings suggest that while JMJD2D is a major contributor to H3K9me3 removal at the IL-12b promoter during infection, other H3K9 demethylases may also participate in this process. Collectively, these data indicate that JMJD2D catalytic activity drives the reduction of repressive H3K9me3 enrichment at the IL-12b promoter, thereby promoting the full induction of IL-12b and other inflammatory genes during *L. monocytogenes* stimulation.

## 4 Discussion

In this study, we identified the histone demethylase JMJD2D as a regulator of host defense against *L. monocytogenes* infection, as summarized in our proposed model (**Figure 7**). Our findings extend the role of JMJD2D in immune regulation by showing that it contributes to macrophage activation and CD8^+^ T cell effector responses during intracellular bacterial infection. Mechanistically, JMJD2D promotes IL-12b transcription through epigenetic and transcriptional mechanisms, including removal of repressive H3K9me3 marks at the IL-12b promoter and cooperation with the NF-κB subunit c-Rel. JMJD2D deficiency was associated with reduced IL-12p70 production and impaired macrophage bactericidal activity, which was partially restored by exogenous IL-12p70. We further found that IL-12p70 promoted IFNγ production by CD8^+^ T cells, whereas IFNγ enhanced macrophage IL-12b expression through the IFNGR2-JAK2-STAT1 pathway. Collectively, these findings support a role for JMJD2D in coordinating macrophage IL-12b/IL-12p70 production and CD8^+^ T cell IFNγ responses during *L. monocytogenes* infection.

**Figure 7:**
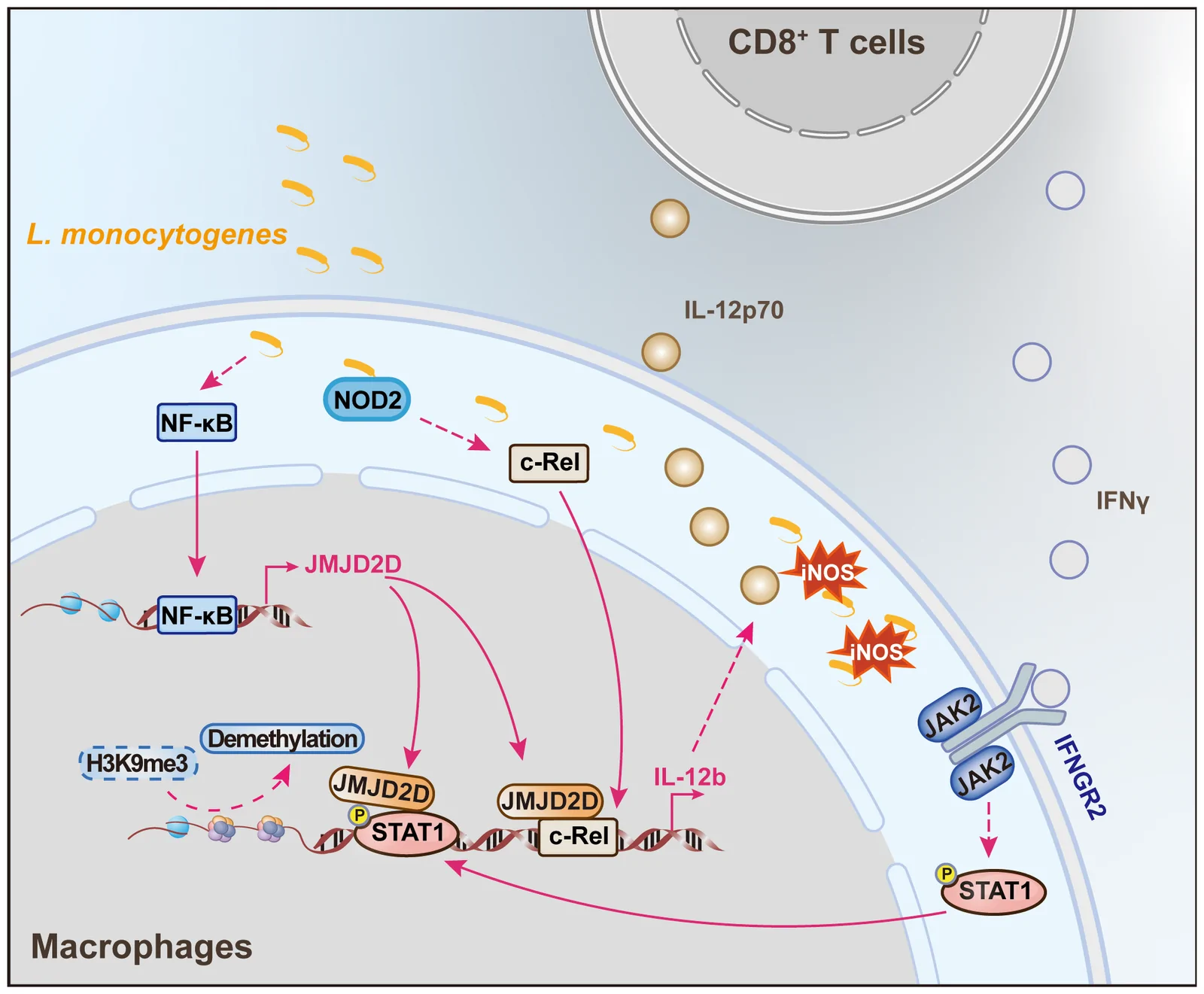
Proposed model of JMJD2D-dependent regulation of macrophage IL-12b production and its association with CD8^+^ T cell IFNγ responses during *L. monocytogenes* infection. JMJD2D enhances host defense through a multi-layered regulatory circuit: 1) In macrophages, JMJD2D is recruited to the IL-12b promoter, where it removes the repressive histone mark H3K9me3 and cooperates with c-Rel to activate transcription; 2) JMJD2D promotes IL-12b expression and IL-12p70 production in macrophages, which is associated with CD8^+^ T cell IFNγ responses; 3) JMJD2D coactivates STAT1 to enhance IFNγ-induced IL-12b transcription through the IFNGR2-JAK2-STAT1 pathway.

Building upon the established role of macrophages as central regulators of host defense against intracellular pathogens, our findings reveal that JMJD2D functions as an infection-responsive epigenetic regulator that coordinates antimicrobial immunity during *L. monocytogenes* infection. The increased susceptibility of JMJD2D-deficient mice to *L. monocytogenes*, together with the unaltered intestinal and splenic architecture under steady-state conditions, suggests that JMJD2D is not broadly required for basal tissue homeostasis but becomes functionally important during infection or inflammatory stress. Consistent with this interpretation, *L. monocytogenes* infection induced JMJD2D expression in macrophages and was accompanied by enhanced p65 phosphorylation, indicating that JMJD2D may function as an inducible component of the early innate immune response. Although heat-killed *L. monocytogenes* was used for the *in vitro* mechanistic studies, this model has been widely adopted for investigating early innate immune signaling because it preserves the major pathogen-associated molecular patterns required for receptor recognition (Shihab et al., 2015; Slonova et al., 2020; Xia et al., 2021). The induction of IL-12b by live and heat-killed bacteria at 4 h post-stimulation supports the use of heat-killed bacteria for these early mechanistic studies, whereas the sustained IL-12b response during live infection likely reflects prolonged stimulation by live bacteria (**Supplementary Figure 5A**). Moreover, live *L. monocytogenes* infection also robustly induced JMJD2D and NOD2 expression *in vivo* (**Supplementary Figures 5B, C**), further supporting the relevance of the identified regulatory mechanisms under physiological infection conditions. Importantly, the functional consequences of JMJD2D deficiency were directly evaluated using live bacteria in intracellular bacterial killing and *in vivo* infection experiments. Beyond IL-12b, JMJD2D deficiency also impaired broader macrophage effector programs, including IL-6 and TNFα production, iNOS expression, and intracellular bacterial killing. These findings suggest that JMJD2D regulates a broader inflammatory and antimicrobial program rather than acting solely as a regulator of a single immune mediator.

Elimination of *L. monocytogenes* depends on effective cellular immunity, in which macrophage activation and intracellular bactericidal mechanisms are critical (Shi and Pamer, 2011; Stavru et al., 2011). Among the macrophage effector programs affected by JMJD2D deficiency, our mechanistic analyses identified IL-12b as an important downstream component of the JMJD2D-dependent response. JMJD2D deficiency markedly reduced IL-12b expression and IL-12p70 production, and impaired macrophage bactericidal activity. Importantly, exogenous IL-12p70 partially restored the bactericidal defect of JMJD2D-deficient macrophages, supporting a functional contribution of IL-12p70 to JMJD2D-dependent antimicrobial activity. The incomplete rescue, however, suggests that IL-12p70 is not sufficient to account for the full antibacterial phenotype, consistent with the broader effects of JMJD2D on macrophage inflammatory and antimicrobial programs. Among the IL-12 family members examined, IL-12b expression was most prominently reduced in JMJD2D-deficient macrophages, whereas IL-12a and IL-23a showed only transient changes (**Supplementary Figures 6A, B**). These findings indicate that impaired IL-12b/IL-12p70 responses represent an important component of the altered macrophage cytokine profile in JMJD2D-deficient mice, although additional JMJD2D-regulated pathways are likely to contribute to the overall host-defense phenotype. In contrast, dendritic cells (CD11c^+^MHCII^+^) and neutrophils (CD11b^+^Ly6G^+^) showed no significant genotype-dependent differences in abundance or IL-12b expression following *L. monocytogenes* infection (**Supplementary Figures 7A–D**). Thus, our data identify the macrophage IL-12b/IL-12p70 response as an important, but not exclusive, component of JMJD2D-dependent host defense, supporting the macrophage as the primary myeloid cell type in which JMJD2D regulates IL-12b/IL-12p70 during early infection.

Given that JMJD2D interacts with the NF-κB subunit c-Rel to promote IL-12b transcription, our findings suggest that JMJD2D functions as an epigenetic co-regulator within the inflammatory transcriptional network controlling IL-12b expression. NF-κB signaling, particularly through c-Rel and p65, is an important regulator of IL-12b transcription during intracellular infection (Sanjabi et al., 2000). Our data further suggest that JMJD2D facilitates c-Rel recruitment to the IL-12b promoter, providing a mechanism linking infection-induced NF-κB activation to epigenetic regulation of IL-12b. This finding is consistent with our previous studies showing that NF-κB signaling induces JMJD2D during TNFα-driven colitis, where JMJD2D promotes tissue recovery and tumorigenesis through the Hedgehog pathway (Zhuo et al., 2020), and that JMJD2D acts as a transcriptional coactivator and histone demethylase to regulate IL-17F-dependent mucosal immunity during *Citrobacter rodentium* infection (Zhang et al., 2024). Thus, the present study extends our previous findings by demonstrating the involvement of JMJD2D in host defense against *L. monocytogenes*, a distinct intracellular Gram-positive pathogen, and by identifying IL-12b as an important downstream target in macrophages.

The regulation of H3K9me3 is a complex process involving both histone methyltransferases and demethylases. Our data show that H3K9me3 levels in infected JMJD2D^-/-^ macrophages were intermediate between those in uninfected WT and infected WT macrophages, indicating that JMJD2D deficiency partially, but not completely, prevented the infection-induced reduction of H3K9me3 at the IL-12b promoter. Consistently, the catalytically inactive JMJD2D-S200M mutant still partially induced IL-12b expression, suggesting that JMJD2D also promotes IL-12b transcription through a demethylase-independent mechanism. Together, these observations indicate that while JMJD2D is a major contributor to H3K9me3 removal at this locus, other H3K9 demethylases, as well as demethylase-independent functions of JMJD2D itself, may also participate in regulating IL-12b expression. Notably, JMJD2D is unique among KDM4 family members in its specificity for H3K9me3, whereas KDM4A/B/C exhibit dual selectivity toward H3K9me3 and H3K36me3 (Krishnan and Trievel, 2013; Hong et al., 2026). Other H3K9 demethylases, including KDM3A, have been shown to regulate macrophage function and inflammatory gene expression (Liu et al., 2019; Qu et al., 2023). Therefore, additional H3K9 demethylases may also contribute to the regulation of H3K9me3 at the IL-12b promoter, and future studies examining their interplay with JMJD2D will be valuable to provide a more comprehensive understanding of H3K9me3 regulation during bacterial infection. More broadly, IL-12b transcription is controlled by multiple transcriptional and epigenetic regulators (Lu et al., 2005; Foltz et al., 2024), and chromatin-modifying enzymes can regulate inflammatory gene expression by altering chromatin accessibility and transcription factor recruitment (Bode et al., 2007; Audu et al., 2022). Therefore, JMJD2D may cooperate with other epigenetic modifiers to establish a transcriptionally permissive IL-12b locus during infection.

Studies using the mouse model of systemic listeriosis have established that innate responses, including macrophage activation and early cytokine production, are essential for initiating adaptive T cell responses and bacterial clearance (D'Orazio, 2019). Consistent with this, our study demonstrates that JMJD2D deficiency impairs CD8^+^ T cell effector function during infection, as evidenced by reduced IFNγ production, despite no significant differences in the proportion or absolute number of splenic CD8^+^ T cells between genotypes. These findings indicate that the reduced IFNγ response in JMJD2D-deficient mice is not due to altered CD8^+^ T cell abundance, but rather reflects impaired accessory signals from macrophages. In support of this, macrophage-derived signals were less effective at inducing CD8^+^ T cell IFNγ production in the absence of JMJD2D, whereas exogenous IL-12p70 was sufficient to enhance this response, pointing to a contribution of the IL-12b/IL-12p70 pathway to CD8^+^ T cell effector function. Nevertheless, additional mechanisms may also contribute to the altered lymphocyte responses observed in JMJD2D-deficient mice. Previous studies have shown that *L. monocytogenes* infection induces type I interferon responses that can promote lymphocyte apoptosis through listeriolysin O (LLO)-dependent mechanisms (Auerbuch et al., 2004; Carrero et al., 2004). Therefore, it is possible that altered type I IFN signaling and/or lymphocyte apoptosis may further influence CD8^+^ T cell survival or responsiveness during infection. Although splenic CD8^+^ T cell abundance was comparable between genotypes, we did not directly assess type I IFN signaling or lymphocyte apoptosis and therefore cannot exclude their contribution to the reduced IFNγ response. This represents a noteworthy limitation of the present study and warrants further investigation. Nevertheless, the reduced IFNγ response induced by CM from JMJD2D-deficient macrophages, together with its enhancement by exogenous IL-12p70, supports a contribution of impaired macrophage-derived cytokine signals. In addition, IL-12 signaling can promote CD8^+^ T cell expansion and survival through IL-2-dependent mechanisms *in vivo* (Starbeck-Miller et al., 2014), suggesting that defective IL-12-mediated communication may affect both effector function and adaptive immune responses during infection.

IL-12p70 is a well-established inducer of IFNγ production and an important link between innate and adaptive immunity (D'Orazio, 2019). In our study, IL-12p70 enhanced IFNγ production by CD8^+^ T cells, whereas recombinant IFNγ increased macrophage IL-12b expression through IFNGR2-JAK2-STAT1 signaling. JMJD2D deficiency impaired IFNGR2 expression and STAT1 activation, suggesting that JMJD2D may also influence the ability of macrophages to respond to T cell-derived IFNγ. Furthermore, IFNγ production by NK (NK1.1^+^CD3^-^) and NKT (NK1.1^+^CD3^+^) cells was comparable between genotypes during the early stage of infection (**Supplementary Figures 8A, B**), supporting CD8^+^ T cells as a key source of IFNγ in the context examined. Together, these observations support a model in which JMJD2D promotes macrophage IL-12b/IL-12p70 production, which favors CD8^+^ T cell IFNγ responses, while IFNγ may in turn reinforce macrophage IL-12b expression. Importantly, CD8^+^ T cell responses were assessed at day 3 after infection, an early stage of immune activation. Thus, our findings primarily support a role for JMJD2D in shaping the early cytokine environment associated with CD8^+^ T cell IFNγ production, rather than establishing its role in sustained antigen-specific immunity. Given that IL-12 signaling can also influence other lymphocyte populations, including CD4^+^ T cells, NK cells, NKT cells, and memory-like CD8^+^ T cells (Bou Ghanem and D'Orazio, 2011; Bou Ghanem et al., 2011; Seregin et al., 2015), the JMJD2D-IL-12b/IL-12p70 axis may have broader effects on immune cell communication during *L. monocytogenes* infection.

Mechanistically, our findings support a model in which JMJD2D may contribute to the coordination of innate and adaptive immune responses through the IL-12b/IL-12p70-IFNγ axis. Following *L. monocytogenes* recognition, NOD2-dependent NF-κB activation promotes c-Rel recruitment to the IL-12b promoter, where JMJD2D facilitates IL-12b transcription through its epigenetic and transcriptional regulatory functions. Exogenous IL-12p70 enhanced IFNγ production by CD8^+^ T cells, while IFNγ increased macrophage IL-12b expression through IFNGR2-JAK2-STAT1 signaling. Interestingly, c-Rel knockdown also affected STAT1 expression, raising the possibility of additional cross-talk between NF-κB- and STAT1-dependent pathways. Other transcriptional and regulatory factors, including IRF4, SOCS1, and SOCS3, may further shape IL-12b expression (Akbari et al., 2014; Chandrakar et al., 2020). Thus, JMJD2D may function as an epigenetic regulatory node that integrates inflammatory signaling with macrophage-T cell communication during intracellular bacterial infection.

Nevertheless, several limitations should be considered when interpreting this model. First, although our data support a functional contribution of the IL-12b/IL-12p70-IFNγ axis, its causal contribution to host protection *in vivo* remains to be established. The partial rescue by exogenous IL-12p70 and the broader inflammatory defects observed in JMJD2D-deficient macrophages further suggest that IL-12b-independent mechanisms may contribute to the overall phenotype. Second, because our *in vivo* studies used global JMJD2D-deficient mice, the relative contributions of macrophages and other immune or non-immune cell types cannot be fully distinguished. Third, CD8^+^ T cell responses were assessed at an early time point, and type I IFN signaling and lymphocyte apoptosis were not directly examined; therefore, these pathways may also contribute to the reduced CD8^+^ T cell IFNγ response. Future studies using cell-specific genetic models, *in vivo* pathway perturbation or rescue, and longitudinal antigen-specific analyses will help further define the causal and cell-type-specific contributions of JMJD2D to host defense.

In summary, our findings support a role for JMJD2D as an epigenetic regulator in host defense against *L. monocytogenes* infection, particularly through its regulation of macrophage IL-12b/IL-12p70 responses. These responses were associated with CD8^+^ T cell IFNγ production, suggesting a potential role for JMJD2D in macrophage-T cell communication during infection. These observations expand our understanding of epigenetic regulation in antibacterial immunity and provide a foundation for exploring JMJD2D-targeted strategies to enhance host antimicrobial responses.

## Data Availability

The original contributions presented in the study are included in the article/**Supplementary Material**. Further inquiries can be directed to the corresponding author.

## Supplementary material

Supplementary Figure 1 JMJD2D is induced upon *L. monocytogenes* infection. **(A)** JMJD2D expression and p-p65 levels were increased in BMDMs following *L. monocytogenes* stimulation. **(B, C)** Densitometric quantification of JMJD2D (**B**, normalized to β-actin) and p-p65 (**C**, normalized to total p65) protein. **(D)** PCR amplification of the *L. monocytogenes*-specific *p60* gene confirmed the identity of bacteria recovered from tissue homogenates. **(E)** Individual body weight trajectories of WT and JMJD2D^-/-^ mice following *L. monocytogenes* infection. Body weight is expressed as percentage of initial body weight at day 0. Each line represents an individual mouse. **(F)** Absolute body weight showed an initial decrease followed by recovery in WT mice, whereas JMJD2D^-/-^ mice exhibited a sustained decrease after infection (*n* = 8). Statistical analysis: **(B, C)** one-way ANOVA followed by Tukey’s multiple-comparisons test; **(E)** two-way ANOVA followed by Šídák’s multiple-comparisons test. ^ns^*p*>0.05; **p* < 0.05.

Supplementary Figure 2 JMJD2D deficiency does not markedly alter splenic lymphocyte abundance during *L. monocytogenes* infection. **(A)** Gating strategy and representative flow cytometry plots for splenic macrophages (F4/80^+^). **(B)** Gating strategy and representative plots for splenic CD4^+^ and CD8^+^ T cells. **(C, D)** The absolute number of splenic CD8^+^ **(D)** and CD4^+^ **(D)** T cells was comparable between WT and JMJD2D^-/-^ mice following infection (*n* = 3). Statistical analysis: **(C, D)** two-way ANOVA with pre-planned Fisher’s LSD comparisons. ^ns^*p*>0.05; ***p* < 0.01.

Supplementary Figure 3 JMJD2D deficiency attenuates inflammatory cytokine and iNOS responses to *L. monocytogenes*. **(A, B)** JMJD2D deficiency attenuated the infection-induced mRNA upregulation of IL-6 **(A)** and TNFα **(B)** in the spleen (*n* = 7). **(C, D)** JMJD2D deficiency attenuated the infection-induced mRNA upregulation of IL-6 **(C)** and TNFα **(D)** in the PMs (*n* = 3). **(E, F)** JMJD2D deficiency attenuated the infection-induced mRNA upregulation of IL-6 **(E)** and TNFα **(F**) in the BMDMs (*n* = 4). **(G)** The mRNA level of iNOS was lower in infected JMJD2D^-/-^ BMDMs than in WT cells (*n* = 6). **(H)** NO production was reduced in infected JMJD2D^-/-^ BMDMs compared with WT cells (*n* = 3). Data are shown as mean ± SEM. Statistical analysis: **(A, B)** two-way ANOVA with pre-planned Fisher’s LSD comparisons; **(C–H)** two-way ANOVA followed by Šídák’s multiple-comparisons test. ^ns^*p*>0.05, **p* < 0.05; ***p* < 0.01; ****p* < 0.001.

Supplementary Figure 4 JMJD2D regulates IL-12b-associated CD8^+^ T cell responses and IFNγ signaling in macrophages. **(A, B)** IL-12p70 stimulation increased IFNγ production by CD8^+^ T cells (*n* = 4). **(C)** Representative flow cytometry plots showing that CM from *L. monocytogenes*-stimulated JMJD2D^-/-^ PMs reduced the frequency of IFNγ^+^ CD8^+^ T cells compared with CM from WT PMs. **(D, E)** JMJD2D deficiency attenuated infection-induced STAT1 phosphorylation in BMDMs **(D)** and PMs **(E)**. Top panels: representative Western blots; bottom panels: densitometric quantification of p-STAT1 normalized to total STAT1 (*n* = 3). **(F)** IFNGR2 expression was reduced in JMJD2D^-/-^ spleens following infection. **(G)** c-Rel knockdown reduced STAT1 expression, whereas STAT1 knockdown did not significantly affect c-Rel expression. Data are shown as mean ± SEM. Statistical analysis: **(B)** one-way ANOVA followed by Tukey’s multiple-comparisons test; **(D–G)** two-way ANOVA with pre-planned Fisher’s LSD comparisons. ^ns^*p*>0.05, **p* < 0.05; ***p* < 0.01; ****p* < 0.001.

Supplementary Figure 5 Comparison of live versus heat-killed *L. monocytogenes* stimulation and *in vivo* induction of JMJD2D and NOD2. **(A)** IL-12b mRNA expression was induced by both live and heat-killed bacteria at this early time point (*n* = 3). **(B)** Infection robustly induced both JMJD2D and NOD2 expression in WT mice, whereas JMJD2D deficiency attenuated NOD2 induction. **(C)** Densitometric quantification of JMJD2D and NOD2 protein levels from **(B)**, normalized to β-actin (*n* = 3). Data are shown as mean ± SEM. Statistical analysis: **(A)** two-way ANOVA followed by Šídák’s multiple-comparisons test; **(C)** two-way ANOVA with pre-planned Fisher’s LSD comparisons. ^ns^*p*>0.05, **p* < 0.05; ***p* < 0.01; ****p* < 0.001.

Supplementary Figure 6 JMJD2D deficiency differentially affects IL-12 family cytokine expression. **(A)** IL-12a mRNA levels in WT and JMJD2D^-/-^ PMs after stimulation (*n* = 3). **(B)** IL-23a mRNA levels in WT and JMJD2D^-/-^ PMs after stimulation (*n* = 3). Data are shown as mean ± SEM. Statistical analysis: **(A, B)** two-way ANOVA followed by Šídák’s multiple-comparisons test. ^ns^*p*>0.05, **p* < 0.05; ****p* < 0.001.

Supplementary Figure 7 JMJD2D deficiency does not alter IL-12b expression in dendritic cells or neutrophils during *L. monocytogenes* infection. **(A, B)** *L. monocytogenes* infection increased the proportions of splenic dendritic cells and neutrophils, with no significant genotype-dependent differences between WT and JMJD2D^-/-^ mice (*n* = 3). **(C, D)** Intracellular IL-12b was not detectably induced in DCs, and inducible in neutrophils, but no genotype differences were observed (*n* = 3). Data are shown as mean ± SEM. Statistical analysis: **(A–D)** two-way ANOVA with pre-planned Fisher’s LSD comparisons. ^ns^*p*>0.05, **p* < 0.05; ***p* < 0.01; ****p* < 0.001.

Supplementary Figure 8 JMJD2D deficiency does not affect early IFNγ production by NK and NKT cells during *L. monocytogenes* infection. **(A, B)** IFNγ production by NK cells (NK1.1^+^CD3^-^) and NKT cells (NK1.1^+^CD3^+^) was comparable between WT and JMJD2D^-/-^ mice during the early stage of infection (*n* = 3). Data are shown as mean ± SEM. Statistical analysis: **(A, B)** two-way ANOVA with pre-planned Fisher’s LSD comparisons. ^ns^*p*>0.05, **p* < 0.05; ***p* < 0.01; ****p* < 0.001.
